# Spinal Dural Arteriovenous Fistula: A Mimic of Demyelinating Disease and Radiculopathy

**DOI:** 10.7759/cureus.24134

**Published:** 2022-04-14

**Authors:** María Alfaro-Olivera, Ricardo D Otiniano-Sifuentes, Lourdes Simbrón-Ribbeck, Laura Zelada-Ríos, Danny Barrientos-Imán, Carlos Abanto, Jorge Ramírez-Quiñones, Ana Valencia

**Affiliations:** 1 Department of Neurovascular Diseases, Instituto Nacional de Ciencias Neurológicas, Lima, PER; 2 Departamento de Diagnóstico por Imágenes, Instituto Nacional de Ciencias Neurológicas, Lima, PER

**Keywords:** multiple sclerosis, radiculopathy, demyelinating disease, epicone syndrome, spinal dural arteriovenous fistula

## Abstract

Spinal dural arteriovenous fistula (SDAVF) is characterized by an abnormal connection between a radicular artery and the venous plexus producing spinal cord venous congestion. It manifests with nonspecific sensory and motor symptoms. We present three cases of SDAVF with different forms of presentation; in two cases, an autoimmune etiology was considered, and in the third case, the initial diagnosis was chronic radiculopathy. In all three cases, a serpentine enhancement was observed after the gadolinium in the dorsal region of the spinal cord corresponded to flow voids in T2-weighted images, which guided the diagnosis. SDAVF should be considered in atypical clinical presentations of radiculopathies or spinal cord syndromes, especially spinal conus or epicone syndrome. Likewise, it should be part of the differential diagnosis of spinal cord presentations of demyelinating diseases such as multiple sclerosis or neuromyelitis optica spectrum disorders.

## Introduction

Spinal dural arteriovenous fistula (SDAVF) is the leading cause of spinal vascular malformation, comprising approximately 70% of cases; however, it is considered a rare pathology that encompasses 3% of all spinal cord injuries [1]. SDAVF is an abnormal connection between a radicular artery and a radicular vein leading to venous congestion, causing nonspecific motor and sensory symptoms that mimic radiculopathies and other entities such as demyelinating or degenerative diseases of the spine, which makes it a condition difficult to diagnose. A careful neurological examination is important since early recognition and timely treatment of SDAVF are essential for a good prognosis [2-4].

We report three cases of SDAVF, highlight their clinical manifestations and errors in the diagnosis, and give suggestions on how to avoid them.

## Case presentation

Case 1

**Figure 1:**
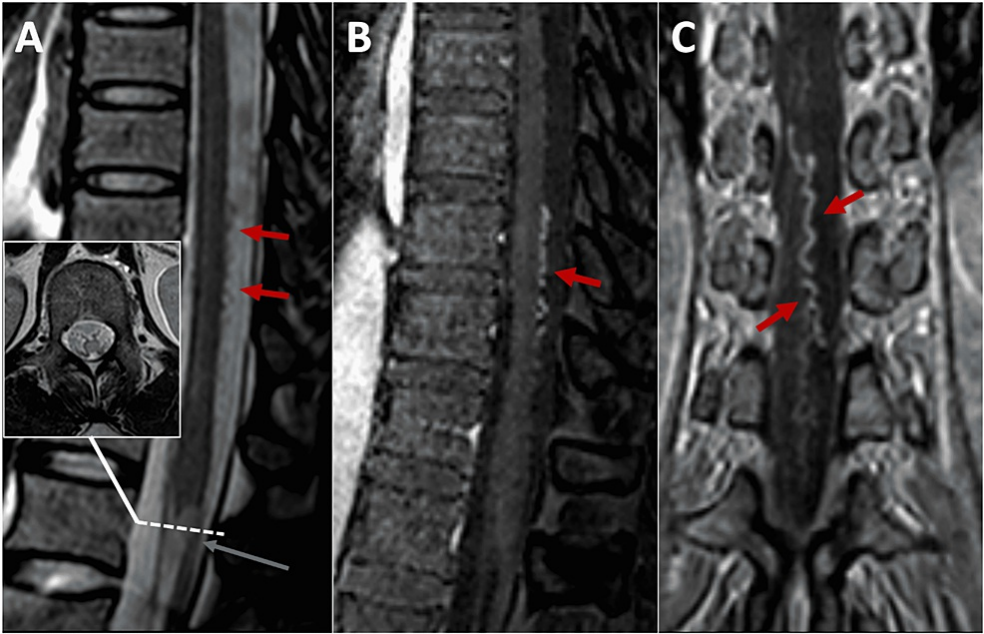
Spinal cord imaging of case 1. (A) T2-weighted sagittal image of the thoracic spine where diffuse hyperintensity is identified in the medullary cone (gray arrow in A), which is confirmed in the axial image. In addition, the flow-void serpentine image on the posterior surface of the spinal cord (red arrows in A). (B) Sagittal and (C) coronal angiographic images showing dilated and tortuous structures on the posterior surface of the spinal cord.

A 29-year-old woman presented to the emergency department with a 10-day history of paresthesia in the perineal region that progressed to the left lower limb; after five days, there was a loss of control of the anal sphincter and severe stabbing lumbar pain radiated to the left lower extremity. The neurological examination showed 4/5 left crural monoparesis with superficial and deep hypoesthesia, left patellar and Achilles hyperreflexia, and negative left plantar reflex. Moreover, she presented with urinary retention and had a left T8 sensory level. Magnetic resonance imaging (MRI) showed diffuse hyperintensity in the medullary cone with homogeneous contrast enhancement (Figure 1A). The autoimmune disorder was considered and methylprednisolone was given for 10 days without significant improvement.

Complete blood count and basic metabolic panel were unremarkable. Cerebrospinal fluid (CSF) showed a glucose level of 58 mg/dl (reference range: 50-80 mg/dl), a protein level of 13.6 mg/dl (reference range: 15-45 mg/dl), and the white cell count was 1 cell/mm^3^. Further MRI investigation showed a faint serpentine contrast enhancement in the posterior part of the spinal cord at T10-T11 levels corresponding to flow gaps in the T2 protocol (Figure 1A). Magnetic resonance angiography (MRA) showed vascular dilations at T10-T12 levels (Figures 1B, 1C).

Case 2

A 45-year-old woman was admitted for a four-year history of relapsing-remitting episodes of weakness in the right lower limb with pressure sensation and burning lumbar pain making standing difficult. Also, she had urinary retention and constipation. All of these symptoms presented within 24 hours. She was treated as transverse myelitis with methylprednisolone and plasmapheresis at a local hospital with partial recovery at discharge. She was able to ambulate at five months without support. A similar episode occurred a year later and she was treated in a similar way, leaving her with slight difficulty in walking. In the last three months before her last admission, she presented intense intermittent low back pain radiating to the right lower limb. Three days before her admission, the clinical picture worsened with persistent "electric shock-like" pain, muscle contractures, and difficulty in urination, so she was brought to our institution.

**Figure 2:**
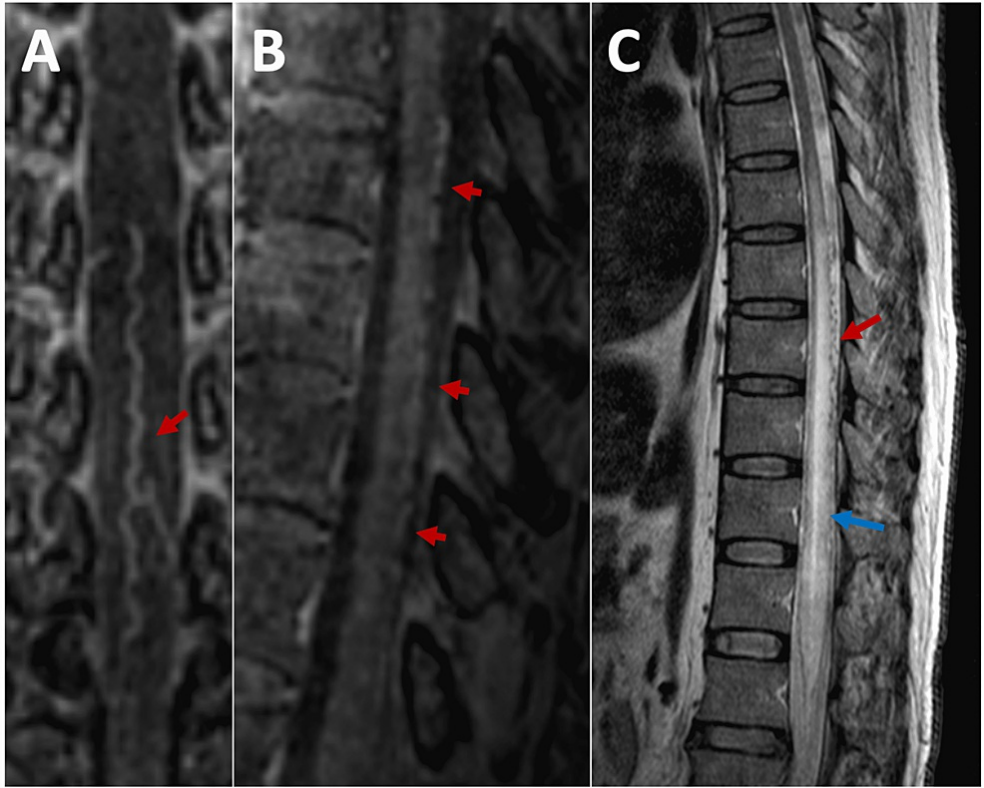
Spinal cord magnetic resonance images of cases 2 and 3. Case 2: (A) Coronal and (B) sagittal contrast images showing dilated and tortuous structures on the dorsal surface of the spinal cord. Case 3: (C) T2-weighted sagittal image showing dorsal myelopathy (blue arrow) and flow-void serpentine images on the dorsal surface of the spinal cord (red arrow).

The neurological examination found 3/5 right lower extremity monoparesis, lower limbs hyperreflexia, positive Babinski, and T4 sensory level. There were no abnormalities in the complete blood count and basic metabolic panel. CSF showed a protein level of 24 mg/dl, a glucose level of 53 mg/dl, and the white cell count was 1 cell/mm^3^; aquaporin-4 (AQP4) antibodies and myelin oligodendrocyte glycoprotein (MOG) antibodies were negative. A demyelinated condition was suspected and she was started on IV methylprednisolone 1 g/day for five days without improvement. Spinal MRI and MRA revealed findings consistent with SDAVF at T8-T11 levels without evidence of myelopathy (Figures 2A, 2B).

Case 3

A 46-year-old man came to the emergency department with a three-year history of lumbar pain and dysesthesia in the lower limbs predominantly at night associated with fluctuating weakness. He was initially cataloged as having bilateral radiculopathy. A year and a half before admission, he presented urinary retention and constipation and presented difficulty in walking 10 months before admission, which progressed until he needed a wheelchair in the last three months.

On physical examination, he had flaccid paraplegia with hyporeflexia in the lower extremities. In addition, he presented muscular atrophy in the lower limbs and T10 sensory level. Complete blood count and basic metabolic panel were unremarkable. Spinal MRI showed extensive myelopathy at the T7-L1 level, as well as tortuous flow void images on the posterior surface, most evident at T8-T10 levels (Figure 2C).

## Discussion

In this case series, we report three different forms of presentation of SDAVF. The first case had an acute onset with a clinical presentation of a medullary cone syndrome, suggesting the diagnosis of transverse myelitis or an anti-MOG disease. In the second case, the diagnosis of multiple sclerosis (MS) was initially raised due to the relapsing-remitting course it had. The third case had a chronic course of radiculopathy that masked the diagnosis until it presented medullary symptoms. As can be seen, the clinical manifestations of SDAVF are varied and nonspecific, leading to a delay in diagnosis and a worse prognosis.

SDAVF predominantly affects middle-aged male patients (with an average age between 55 and 60 years) [4], which contrast with our cases, which are preferably women with an average age of 40 years (26-45 years). In our cases, the location of the SDAVFs was at the T8-T12 level, a location that occurs more frequently (70-80%) [4]. The time from the onset of clinical symptoms to diagnosis ranges from 11 to 27 months, according to the reported series [5], although there may be acute presentations, such as case 1. According to the Spetzler classification [6], SDAVFs are of low flow, causing venous congestion in the most caudal regions of the spinal cord. However, mechanisms of vascular compression have also been proposed as the cause of some radicular syndromes [2].

Due to the low specificity of the symptoms of SDAVF, this entity is underdiagnosed and 78% misdiagnosed [3]. The most frequent diagnostic errors are lumbar spinal stenosis, transverse myelitis, prostate disease, and intramedullary tumors [3,7]. Less frequently they are misclassified as MS or neuromyelitis optica spectrum disorders (NMOSD), as in two of our cases [3,8]. Despite this, there are clinical symptoms and syndromes that may suggest SDAVF. The most common presentation includes a gradual progression of difficulty in walking, leg paresthesia, sensory loss, and radicular pain. All these symptoms were present in the three cases that we report; however, they differed in the form of onset and course of the disease. Furthermore, sphincter dysfunction was also a common symptom in all three cases. Its frequency is 24.5% and it usually appears late [7,9]. An important symptom is a lumbar or radicular pain that has been described in more than half of the cases of SDAVF [4,5] and places it within the differential diagnosis of painful myelopathy along with other demyelinating diseases. By encompassing symptoms in clinical syndromes, SDAVF frequently presents as a radicular, medullary cone, or epicone syndrome [2-4]. Therefore, when faced with these syndromes, this entity should always be considered.

In 90% of SDAVF cases, there is a central medullary hypersignal on T2-weighted images, which is caused by caudal venous congestion. This causes the medullary cone to be compromised in up to 80% of SDAVF [4]. In our cases, two had evidence of myelopathy, both with a compromise of the medullary cone; one of them had an extension of seven vertebral segments. Therefore, SDAVF should be part of the differential diagnosis of longitudinally extensive myelitis [10,11], since they tend to have an extension of five to seven vertebral segments on average [4]. In our cases, myelopathy initially suggested anti-MOG disease and neuromyelitis optica. However, the flow voids observed in the dorsal surface of the spinal cord in the T2-weighted images suggested the diagnosis of SDAVF. This finding represents tortuous and dilated veins and reaches a specificity of 97% together with the hypersignal in T2, as described previously [12]. A less frequent finding (79%) that we were able to observe in all our cases is peripheral enhancement [7]. With these findings, we performed MRA, which can show serpentine flow in perimedular structures in up to 100% of cases, and indicate the level of the lesion [13]. Catheter angiography is the gold standard; however, it has limitations. Sometimes it is not possible to detect it, usually due to factors related to the operator. In our cases, it could not be detected due to a lack of adequate catheters and therefore surgical treatment was offered [8].

## Conclusions

SDAVF should be considered in atypical clinical presentations of radiculopathies or spinal cord syndromes, especially in spinal cone or epicone syndrome. Also, it should be part of the differential diagnosis of spinal cord presentations of demyelinating diseases such as MS or NMOSD. Flow voids on T2-weighted MRI within the subarachnoid space that correspond to areas of gadolinium enhancement are specific findings of this entity. Given this finding, confirmatory angiographic studies should be requested.
